# A Convenient and Delicate Test for Albumen

**Published:** 1883-02

**Authors:** 


A Convenient and Delicate Test for Albumen.—Dr. A. W. Abbott, of Minneapolis, Minn., sends us the following description of an easy and delicate method of testing albumen:

"Pour a few drops of urine gently down the inside of a glass vessel containing acidulated water at the boiling point. If albumen be present a more or less dense cloud will form just at the dividing line between the fluid tested and the clear water above. As the contrast in opacity is between the clear water and the milky albuminous cloud, the test is very delicate, one-twentieth of one per cent. of albumen making a very perceptible clouding. It has all the advantages of the ordinary heat and acid test and Heller's nitric acid test. It is even better than the latter in a cloudy fluid as in urine, with urates in excess, because the clear water above makes a perfect medium in which to detect the faintest cloud, while the layer of coagulated albumen in Heller's test may be entirely obscured by the opacities in the fluid itself. If no test-tube or nitric acid is at hand, pour boiling water into a common tumbler, let it stand a moment to insure the heating of the bottom of the tumbler, empty, refill, acidulate with vinegar, and proceed as before. While this is a modification of the heat and acid test it has the advantage of being applicable under all possible circumstances, whether special apparatus is at hand or not. It is as convenient and accurate in the farm-house as in the laboratory.—*Medical Record.*

